# Best practice guidance for recreational and professional drones near colonial breeding birds

**DOI:** 10.1371/journal.pone.0332619

**Published:** 2025-11-05

**Authors:** Estefania Velilla, Nadia Hijner, Annelies van Ginkel, Maarten Zwarts, Jannes H. T. Heusinkveld, Kees Koffijberg, Kees Oosterbeek, Julia Stahl, Sjoerd Duijns, Laura L. Govers

**Affiliations:** 1 Marine Conservation Group, Groningen Institute for Evolutionary Life Sciences, University of Groningen, Groningen, The Netherlands; 2 Wageningen Marine Research, Wageningen University & Research, Yerseke, The Netherlands; 3 The Fieldwork Company, Groningen, The Netherlands; 4 Sovon, Dutch Centre for Field Ornithology, Nijmegen, The Netherlands; 5 Department of Coastal Systems, Royal Netherlands Institute for Sea Research, Texel, The Netherlands; Universidad Miguel Hernandez de Elche, SPAIN

## Abstract

Drone use has increased sharply worldwide over the past decade, leading to more frequent interactions with wildlife. The rapid advancement of drones for ecological monitoring and research has further contributed to these encounters, which may disturb animal behavior, such as triggering flight responses in birds. Therefore, best-practice guidelines are urgently needed to help operators and site managers minimize disturbances. This study aimed to establish safe operating distances for seven common colonial breeding bird species: black-headed gull (*Chroicocephalus ridibundus*), herring gull (*Larus argentatus*), lesser black-backed gull (*Larus fuscus*), Sandwich tern (*Thalasseus sandvicensis*), common tern (*Sterna hirundo*), Eurasian spoonbill (*Platalea leucorodia*), and great cormorant (*Phalacrocorax carbo*). We assessed the effects of professional and consumer-grade drones flying at altitudes between 5 and 50 meters on the flight responses of these species at breeding sites in the Dutch Wadden Sea. Of 1492 drone flights, 7.4% caused disturbances, defined as more than 10% of birds becoming airborne. Flight initiation distance (FID), the distance between a bird and the drone at the moment of flight response, varied by species. Sandwich terns and common terns had the largest FID (>170 m), followed by black-headed gulls (>160 m), herring gulls and lesser black-backed gulls (>60 m), while great cormorants and Eurasian spoonbills had the shortest (~5 m). When selecting drone flight locations, we recommend considering species-specific FID and using the maximum diagonal FID as a guideline. Disturbance decreases with altitude, so flights should be conducted at 50 meters or higher whenever possible. These findings provide concrete guidelines to inform policy and promote the responsible use of drones in wildlife research and management.

## Introduction

Unmanned aerial vehicles (UAS), or drones, have increased in popularity over the past years for recreational and professional purposes [1,2]. Studies on the use of drones for ecological research and monitoring have shown that there are many ways research and nature management can benefit from their use, such as, facilitating aerial views, safer access to difficult-to-reach areas, user-friendly, relatively inexpensive, higher survey revisit periods and low altitude flights [3–6]. However, drones can also disturb wildlife, as has been shown by several studies such as, [2], which shows an increase in disturbance in sub-Antarctic seabirds, [7] who provide evidence that drones flown lower than 60m cause disturbance in several terrestrial mammals, or [8] who report that marine mammals exhibited a fleeing response to drone flights. Understanding the effects of drones on animals is therefore crucial to further develop the use of this tool in a research and monitoring context or to responsibly enjoy it as a recreational tool [9].

In an ecological context, disturbance refers to a deviation from a baseline behavioral state caused by an external agent, which can disrupt populations, communities or ecosystems [10,11]. While there is sufficient scientific evidence that drones can disturb wildlife, there is a considerable variation in the extent of this response (disturbance) [2,12,13]. For example, [12] found little to no behavioral response to drones in blue whales, while [13] found that bottlenose dolphins significantly changed their behavior, reorienting in response to drones. Here, we focus on birds, which are very sensitive to drone disturbances [1,14]. Behavioral responses depend on several factors: species, life-history stage, habitat type, drone type, and flying characteristics such as speed and distance [1]. In birds, particularly during the breeding season, disturbance has been defined behaviorally as flushing (i.e., taking flight), increased vigilance, altered parental care or even nest abandonment in response to perceived threats [14]. A recent meta-analysis [15] has identified a set of consistent disturbance behaviors in response to drone activity, especially in nesting birds. These behaviors include alert postures, mobbing, increased vocalization, flushing, and reduced time spent on the nest. Among these, flushing is one of the most visible and frequently used indicators in field assessments.

As drone-based surveys are increasingly used to monitor nest counts and breeding success, documenting responses and response distances for the different species of breeding birds under changing circumstances (e.g., drone type and flight altitude) is crucial to support the establishment of guidelines for the safe operation of drones and for nature management. Current regulations and guidelines for the operation of drones in the EU are anthropocentric and primarily focused on the safety and privacy of people and piloted aircraft [16,17]. For instance, in Europe, the European Union Aviation Safety Agency (EASA) oversees drone regulations across member states [18]. Key regulations are human-centred, including guidelines such as no flying over crowds, maintaining a visual line of sight and no-fly zones near airports [19]. Wildlife is only indirectly covered in the regulations, as flying over Natura 2000 sites and protected areas may be restricted and require authorization [19,20]. Regulations in the United States (U.S.) and Canada, on the other hand, are more specific about nature and include a ban on flying over National Parks and National Wildlife Refuges to prevent wildlife disturbance. Additionally, U.S. and Canada regulations prohibit drone flights near marine mammals under the Marine Mammal Protection Act [19]. It is evident that the Netherlands and the E.U. in general fail to seriously consider wildlife in their drone-use regulations [19], calling for attention to the effect of drone-wildlife interactions and the inclusion of wildlife-centerd guidelines. Especially as the use of drones increases in ecology [14].

Although the use of drones has been integrated into conservation research, monitoring, and nature management relatively fast, standardized best practice guidance is lacking, causing policy to lag behind. This may lead to unregulated drone use by both professional practitioners and recreational flyers. Furthermore, many bird species across Europe and North America are strongly protected by several laws: the Bird Directive, the Habitat Directive, the Bonn Convention of Migratory Species (Europe), the Migratory Bird Treaty Act, the Endangered Species Act, and the North American Bird Conservation Initiative (North America). Guidelines for safe use of drones, both for management purposes and the occasional recreational flyer, are needed to ensure effective protection of vulnerable bird species and habitats. Therefore, in this study, we provide the evidence base for the development of management guidelines and policy recommendations for the safe operation of drones over or around breeding bird colonies. Moreover, we find it important to compare the disturbance effects of consumer-grade drones vs. professional drones, as these can differ greatly in size and weight (see methods section), and therefore, probably in how they are perceived as a threat [14]. In spite of these differences, only a couple of studies have tested the effect of drone size on disturbance [14], and their results are contrasting. One study showed that smaller drone models caused less disturbance [21], while [22] found the opposite trend, both of them using rotary-wing drones.

The Wadden Sea is a global hotspot for migratory shorebirds, with many species using this World Heritage Area as a stopover site or as breeding grounds. The Wadden Sea has recently become a bottleneck for protected colonial breeding birds such as terns, gulls and pied avocet [23]. Breeding success in the Wadden Sea, however, is low and breeding site availability and suitability seems limited. Monitoring of (colonial) breeding birds in the Wadden Sea has been carried out since 1991 [24], where fieldwork is carried out by a large number of volunteers and staff from various nature conservation agencies and local site managers. This fieldwork is time-consuming, expensive and labor-intensive, and it may be vulnerable to observer bias. Moreover, it can be difficult in remote and difficult-to-reach locations. By using drones, the full research/ monitoring area can be more rapidly covered, difficult-to-access sites become more easily accessible, and it can be cheaper [14]. Although the effect of drones on the disturbance behavior of wildlife is increasingly being studied (including birds), the effects can be taxa- and location-specific [16,25,26], and evidence-based best practice guidelines for nature management are rare.

The aim of this study was twofold. First, we aimed to estimate disturbance distances for the 7 most common colonial breeding bird species in the Wadden Sea to provide tools for users, nature managers, and policymakers to define ‘safe’ operating drone distances. Second, we aimed to investigate the behavioral effects of professional vs. consumer-grade drones and flight altitude on the same species and locations. In our study, we used a behavioral threshold to define disturbance: a colony was considered disturbed when more than 10% of the birds became airborne (i.e., > 10% flushing) simultaneously in response to an approaching drone. This threshold is was based on observed background activity levels and aims to differentiate between normal colony dynamics and drone-induced responses.

## Methods

### Study sites and study species

The Wadden Sea (a UNESCO World Heritage Site of 10.000 km^2^) is an intertidal ecosystem, with tidal mudflats and salt marshes stretching along the northern coastlines of the Netherlands, Germany, to the west coast of Denmark [27,28]. It is considered one of the most important coastal wetlands and the largest intertidal ecosystem worldwide [28,29]. It is a globally important area for breeding birds and a wintering site and stopover for migratory bird species [27,30].

Here, we quantified the potential disturbance effect of two different types of drones on seven colonial breeding bird species common in the Wadden Sea: common tern (*Sterna hirundo*), Sandwich tern (*Thalasseus sandvicensis*), black-headed gull (*Chroicocephalus ridibundus*), Eurasian spoonbill (*Platalea leucorodia*) and the great cormorant (*Phalacrocorax carbo*), and mixed colonies of herring gull (*Larus argentatus*) and lesser black-backed gull (*Larus fuscus*). We selected these seven species because they encompassed all relevant colonial breeding shorebirds present in the area except for Arctic tern (which had become too rare to include in this study) and pied avocet (*Recurvirostra avosetta*). All of the species we selected, except for spoonbill, are currently declining in both numbers and breeding success in the international Wadden Sea [31]. In addition, the selected bird species represent a diverse selection of coastal birds with a global distribution and ecological key roles as avian mesopredators: e.g., tern, gull, cormorant and spoonbill species. Moreover, in this study, we focused on colonial species rather than solitary breeding birds, as behavioral disturbance effects of colonial breeding birds may cascade through colonies and thus have stronger effects on a population level.

**Fig 1:**
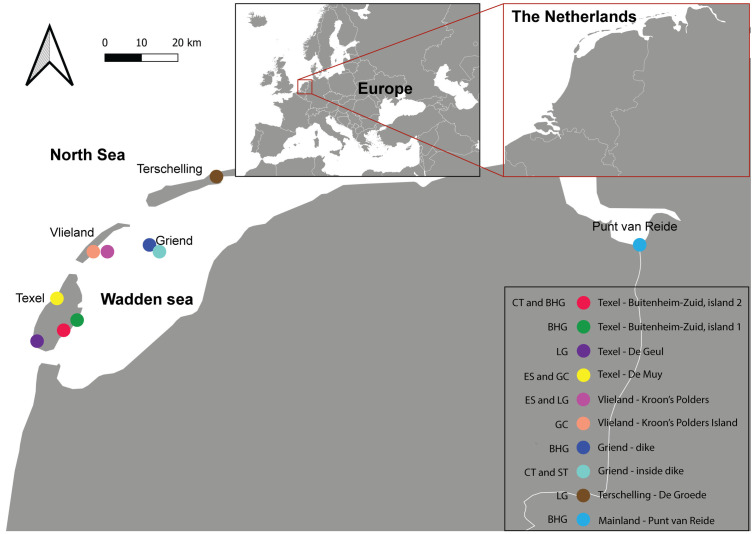
This figure indicates the location of The Netherlands within Europe; a map of The Netherlands; and the location of the colonies used in this study. The abbreviations of the species are as follows: CT: common tern; BHG: black-headed gull; LG: large gulls; ES: Eurasian spoonbill; GC: great cormorant; ST: Sandwich tern. Maps were made with Natural Earth. See S1 Table for more detailed information about each colony.

**Fig 2:**
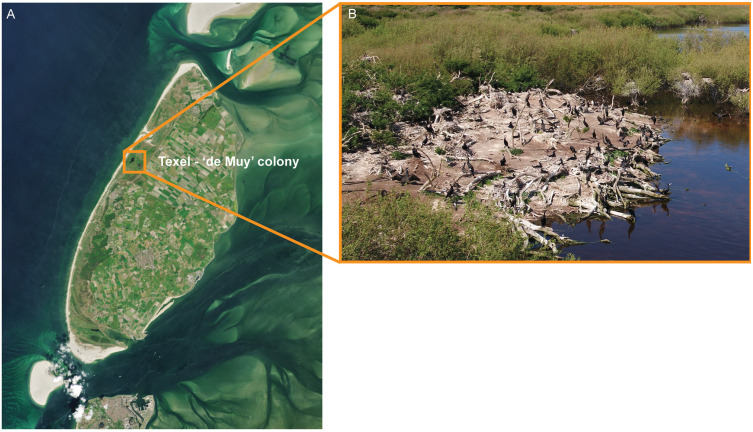
A) Location of a cormorant and Eurasian spoonbill colony on the island of Texel. B) a zoomed-in image of the location of the colony. Landsat satellite imagery courtesy of NASA Goddard Space Flight Center and U.S. Geological Survey. Close up photo of a cormorant colony made during our disturbance experiments with drone DJI Matrice 210 RTK, equipped with a Zenmuse X5s camera.

To study these seven species, we chose ten different breeding colonies that varied in species composition, numbers, and local characteristics (S1 Table, Fig 1 and see Fig 2 for example of a great cormorant *P. carbo* and Eurasian spoonbill *P. leucorodia* colony). Six were single-species colonies, and 4 were mixed colonies (S1 Table). The European herring gull and the lesser black-backed gull were indistinguishable in our study areas and are thus combined and hereafter referred to as ‘large gulls’. In our analysis, we have generalized the behavioral response to our experimental treatments across colonies and have not distinguished the effects for monospecific (single) and multi-species (mixed) colonies. This is because almost none of the species from our study occurred in both single and mixed colonies, with the exception of large gulls, for which we did test the effect of colony composition. The number of colonies included in this study was limited both by available colonies in the study area and by the time available in the breeding season; We wanted to ensure that all flights were carried out within a two-week time frame to maximize alignment of the breeding phase of all colonies (e.g., late egg phase, before hatching).

### Defining disturbance

In an ecological context, disturbance can be defined as an agent, force, or process that causes perturbation (including stress) in an ecological component or system relative to a reference state [11]. For this study, we defined disturbance as the deviation from a baseline behavioral state, e.g., nesting or resting, to a flight status. In the context of breeding birds, the behavioral unwanted state that we monitored entailed leaving the nest or its vicinity (going airborne) in response to an approaching drone. Although we focused on a behavioral response, another reaction to drone disturbance could be physiological stress, which we have not quantified.

To determine the point at which we classified a colony as disturbed, we observed the different colonies for 4–8 hours, noting every 5 minutes the % of birds that were airborne above the colony without noticeable disturbance factors, e.g., birds of prey or people in the area. We used this background reference level as a baseline for a normal state or undisturbed colony by rounding off general activity levels. Our results showed that the mean percentage of birds in the air without disturbance factors ranged between 1.1–8.8% (see S1 Text for full protocol on determining baseline behavior and S2 Table for detailed results on baseline behavior). Subsequently, we concluded that <10% of birds in the air at any given moment can be defined as ‘undisturbed’, and that disturbance could be defined as >10% of airborne birds in a breeding colony (or the monitored zone within a colony) in response to our approaching drone. We classified disturbance intensity into four categories: < 10%, 10–50%, 50–75% and 75–100%. Each category represents the percentage of the colony that is airborne due to the presence of the drone.

### Drone characteristics and colony exploration

Using two types of multirotor drones: a consumer drone DJI Air 2S (diagonal size unfolded: 253 × 183 × 77 mm, color gray, weight 595 g, max speed 6 m/s, equipped with a built in 20mp camera), and a professional-grade drone DJI Matrice 210 RTK (diagonal size unfolded 887 × 880 × 408 mm, color black, weight 3.8 kg, max speed 23 m/s, equipped with a Zenmuse X5s camera) we made explorative flights to first determine the size and distribution of the colony. During this explorative phase, we also selected the best observation point to see the entire colony. When the colony was too large to be monitored from one observation point, we divided it into monitoring (“observable”) zones. Zonation of observations was necessary in large colonies to ensure we could observe colony behavior unobstructed. Zones were delineated using either natural visual landscape features such as separate breeding islands or by using natural markers such as notable shrubs. We have included only the first observation zone in our analysis, avoiding cascading disturbance events that could have been caused by disturbance in the first zone. Furthermore, we estimated the maximum number of breeding birds in the colonies and zones using the footage of the DJI Matrice 210 RTK. This allowed us to estimate the percentage of the colony disturbed during the flights. We used several methods to estimate the number of breeding birds, depending on the density and spatial distribution and what nature managers permitted. In some colonies, we counted the number of breeding birds from a distance with telescopes. In other colonies, colony size estimation was done by means of an observer walking through the colony counting the nests. Another technique we employed was flying at an altitude of 50 m over the colonies with the DJI M210 drone and counting breeding birds based on footage.

### Drone disturbance experiments

For the controlled disturbance experiments, we conducted flights with a consumer drone, DJI Air 2S and a professional-grade drone, DJI Matrice 210 RTK while concurrently recording behavioral reactions on video. Flights were conducted in a “target-oriented” manner, which meant approaching the colony horizontally at 5-, 10-, 15-, 20-, 30-, 40-, and 50 m altitude, randomizing the order of the altitude, and the drone type that was first used (S1 Fig). The flight speed of the drone when approaching colonies was 5 m/s. The take-off distance of the drones varied per colony (85 m – 420 m; see S1 Table for all take-off distances), depending on the characteristics of the colony and the line of sight. Time elapsed between the landing of a flight and the next flight was approximately 10 minutes, which was the time needed to get the equipment ready for the next flight. Behavioral reactions were tallied continuously per flight, and a disturbance score was assessed for each trial, including the start and end times of disturbances.

When a flight caused a disturbance event (>10% of birds from the colony airborne), consecutive trials continued only for altitudes higher than the one that caused the disturbance. After a disturbance was observed during a flight, the pilot would finish the flight as planned and subsequently return the drone to the take-off point. The next planned flight was only commenced after all birds had settled back into the colony to a non-disturbance state (<10% airborne) for at least 10 minutes. If no disturbance was scored in the consecutive trials, the altitude that caused the disturbance was tested again. Thus, the randomization of the altitudes sometimes influenced the number of trials we could conduct on a given day in a particular colony. The first flight of a series was never done at 5 m to avoid excessive disturbance that could cascade to the flights at higher altitudes. Per day, we flew a maximum of 8 flight series over a colony. A flight series consisted of a set of a maximum of 7 flights (one per altitude category) flown with one of the drone types. Drone type was randomized across the different series (see S3 Table for an example of a flight series). Each colony was revisited for at least two days to reach 6–8 flight series per drone type per colony. Flights were carried out during daylight hours between May 19 and June 11, 2021, in the breeding season, before the egg-hatching phase.

### Calculating horizontal and diagonal flight initiation distance

**Fig 3:**
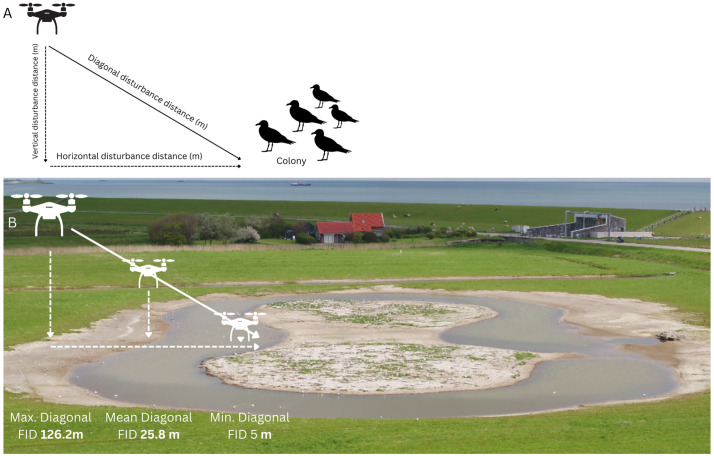
A) illustration of horizontal and diagonal flight initiation distances (FID) in meters, in addition to altitude in meters in a hypothetical colony. B) an example of maximum, mean and minimum diagonal FID (m) at a black-headed gull colony. The image in this example (B) is a screenshot of a video recording made during our disturbance experiments with drone DJI Matrice 210 RTK, equipped with a Zenmuse X5s camera.


The distance at which a bird flees from a perceived threat (a drone, in our case) is defined as the flight initiation distance (FID) [31]. Since we could not measure the distance between the position of the drone and the exact location of every bird, we calculated the horizontal and diagonal distance between the location of the drone when a disturbance was scored and the nearest edge of the colony (Fig 3). We used this distance as a proxy for actual flight initiation distances and as our measure of disturbance. We noted when a disturbance occurred, and we extracted the coordinates of the disturbance source (the drone) at the time of disturbance initiation. Diagonal FID was calculated using the Pythagorean theorem [32] (Fig 3). Horizontal distance, which is needed to calculate diagonal FID, was measured by calculating the horizontal distance of the drone (based on the coordinates during the onset of the disturbance) to the nearest edge of the colony in QGIS using the NNJoin plugin (QGIS Development Team, version 3.24.3-Tisler). With the horizontal distance we can estimate the bare minimum distance to prevent disturbance in a breeding colony, and it is easier to implement in management. Diagonal distance incorporates both the horizontal distance and the altitude. Diagonal distance can be used to calculate the angle of approach.

$$Diagonal\;distance=\;\sqrt{{Horizontal\;distance}^{\text{2}}+{Drone\;flight\;altitude}^{\text{2}}}$$

### Statistical analysis

#### Effect of species, flight altitude and drone type on percentage of disturbance.

All statistical analyses were done with R version 3.3.1 (R Core Team, 2016), run in the RStudio interface (version 2022.07.01). We calculated the percentage of disturbance observations by adding the number of disturbance observations per disturbance intensity and dividing it by the number of flights, per drone type, and per flight altitude.


$$\%\;disturbance\;observations=\;\frac{\sum \;disturbance\;observations}{\sum \;flights\;per\;drone\;type,\;per\;altitude\;}$$

Then, to analyze whether percentage disturbance was significantly different between species, we used a Kruskal-Wallis test, followed by a pairwise Wilcox test with Bonferroni correction for multiple testing. Moreover, we used a Generalized Linear Mixed Effects Model (glmer in the R package “Lme4”) with binomial distribution to test the interaction between species and altitude, and to test the effect of drone type. We included colony and day of flight as random effects in our model. We compared our model to a null model containing only the intercept and the random effects with the Akaike Information Criterion (AIC) as a model fit metric. Additionally, we ran separate Kruskal-Wallis tests for each species to test the effect of drone type. We did this, because our mixed effect models showed a significant result of drone type on disturbance. However, since drone type was not balanced in our experimental design for all species and altitudes, we decided to test its effect in the individual species.

### Effect of flight altitude on intensity of disturbance

To determine the effect of altitude on the disturbance intensity, we used multinomial regression, using the function “multinom” from the R package “nnet” [33]. We included altitude as a discrete variable, interacting with species. We merged drone type for this analysis, as drone type did not have a significant affect an earlier version of the multinomial regressions. As the multinomial regression output provides the log odds of events, we calculated the odds ratio by taking the exponential of the coefficients and a separate model for each species was used. We report Confidence Intervals (CI 2.5% and 97.5%).

### Effect of species and drone type on diagonal flight initiation distance

To investigate differences in flight initiation distance (FID) between species and drone types, we opted for a Kruskal-Wallis test and pairwise Wilcox test with Bonferroni correction for multiple testing. We used non-parametric tests because (generalized) linear models did not meet normality model assumptions and the diagnostics of the generalized linear models showed that the model did not fit the data well. Furthermore, to test whether there was a relationship between FID and disturbance intensity, a Kruskal-Wallis test was performed per species. Because of the high correlation between horizontal and diagonal FID (R^2^ = 0.94), we only report statistics for diagonal FID, which we consider to be the most complete. However, we also provide the horizontal FID as it is an easier distance to calculate for drone users and managers.

### Differences in flight initiation distance between mixed and single species colonies

Differences in diagonal FID between mixed- and single species colonies could only be tested for large gulls, as these were the only species from our study that occurred in mixed and single species colonies, and which displayed disturbance behavior in both types of colonies. To test differences in horizontal and diagonal FID, we used a Kruskal-Wallis test.

## Results

**Fig 4:**
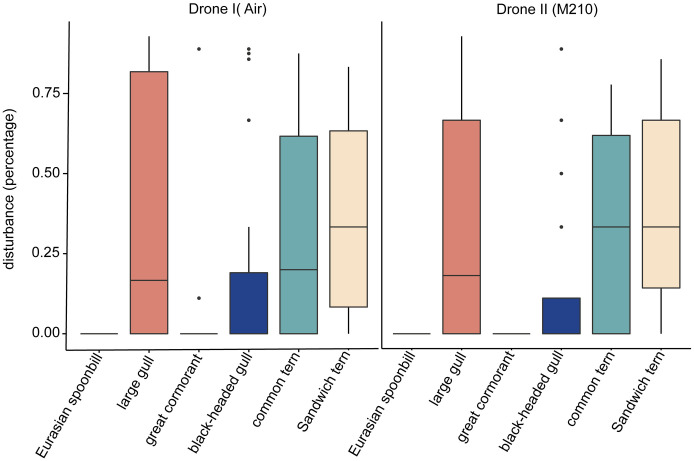
Fraction of observations in which disturbance was recorded per species, per drone type.

We carried out a total of 1492 flights across 7 species on 10 colonies (Table 1). Although 7 species were monitored, herring gull and lesser black-backed gull are treated as one species. Therefore, the results are shown for 6 species. Disturbance occurred on 7.4% of all flights (111/1492 flights). Disturbance differed significantly between species (Kruskal-Wallis, *df* = 5, χ^2^ = 65.736, p < 0.01, Fig 4). See S4 Table in the supplementary materials for pairwise comparisons.

**Table 1:** Number of drone flights per species and colony and percentage of disturbance observations. Fewer flights were conducted for disturbance-sensitive species, as operations stopped when disturbance was recorded.

Species	Colony	nº Flights	nº Disturbed flights	Disturbance per species, per colony (%)	Overall disturbance per species (%)
black headed gull	Texel, Buitenheim-Zuid, island 2	103	0	0%	5.6%
	Texel, Buitenheim-Zuid, island 1	113	2	1.7%	
	Griend, dike	15	5	33.3%	
	Mainland – Punt van Reide	108	12	11.1%	
large gulls	Texel – De Geul	132	28	21.2%	12.1%
	Terschelling – De Groede	100	6	6%	
	Vlieland – Kroon’s Polders	173	15	8.7%	
common tern	Texel – Buitenheim-Zuid, island 2	103	6	5.8%	16.1%
	Griend – inside dike	77	23	29.8%	
Sandwich tern	Griend – inside dike	77	13	16.8%	16.9%
great cormorant	Vlieland – Kroon’s Polders island	113	1	0.9%	0.5%
	Texel – De Muy	101	0	0%	
Eurasian spoonbill	Vlieland – Kroon’s Polders	176	0	0%	0%
	Texel – De Muy	101	0	0%	

**Fig 5:**
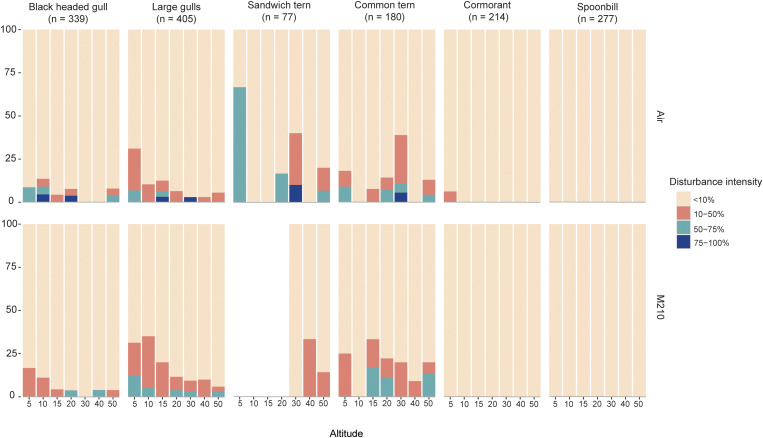
Percentage of disturbance observations in which a particular disturbance intensity was observed, per species, drone type and drone flight altitude. Empty (not colored) bars mean that altitude was not tested.

No disturbances occurred in Eurasian spoonbill colonies (0%, N flights = 277, Table 1, Fig 4). In contrast, large gulls, Sandwich terns, and common terns were most sensitive to disturbance (Table 1, Fig 4). The latter is most likely an underestimation of sensitivity to drone disturbance since fewer flights were completed for disturbance-sensitive species since flights at low(er) altitudes were omitted after re-occurring disturbance (see methods section). Generally, 10–50% of the colony showed take-off behavior when disturbance occurred. (Table 2, Fig 5).

**Table 2:** Percentage of observations per disturbance intensity, per species.

Species	<10%	10-50%	50-75%	75-100%
black-headed gull	94.4	3.2	1.8	0.6
large gulls	87.8	9.5	2.2	0.5
Sandwich tern	83.1	10.4	5.2	1.3
common tern	83.9	10.6	5	0.5
great cormorant	99.5	0.5	0	0
Eurasian spoonbill	100	0	0	0

### Effect of species, flight altitude and drone type on disturbance and disturbance intensity.

We found a significant relationship between species and flight altitude for the Sandwich tern (GLMM, z = −2.48, p = 0.01), common tern (z = −1.95, p = 0.05), black-headed gull (z = −2.82, p = 0.004) and large gulls (z = −3.93, p < 0.001). Compared to a null model, our full model left the least variation unexplained (∆ AIC: 37.7). While drone type appeared as significant in our mixed model, we concluded that species-specific models were needed to test the effect of drone type, as drone type was not balanced in our experimental design (see S2 Fig.). The species-specific models show that drone type did not have a significant effect on any of the species. Moreover, we found no significant differences in the overall disturbance between consumer and professional drone types (Kruskal-Wallis, *df* = 1, χ^2^ = 0.248, p = 0.61, Fig 4). Therefore, we decided to merge the data from both types of drones for the rest of the analysis.

Furthermore, we found a significant effect of the relationship between species and altitude on disturbance, for a disturbance intensity of 10–50% (black headed gull, z = −3.81, std, err: 0.018, p < 0.001; large gulls, z = −2.75, std. err: 0.010, p = 0.005; great cormorant, z = −2.21, std. err: 0.17, p = 0.02, Fig 5, S5 Table) and for disturbance intensity of 50–75% (black-headed gull, z = −2.22, std, err: 0.022, p = 0.02; large gulls, z = −2.36, std. err: 0.02. p = 0.01, Fig 5, S5 Table). There was no significant interaction between species and altitude at disturbance intensity of 75–100%. This is most likely due to rarely encountering this level of disturbance intensity.

### Safe drone operating distances

**Fig 6:**
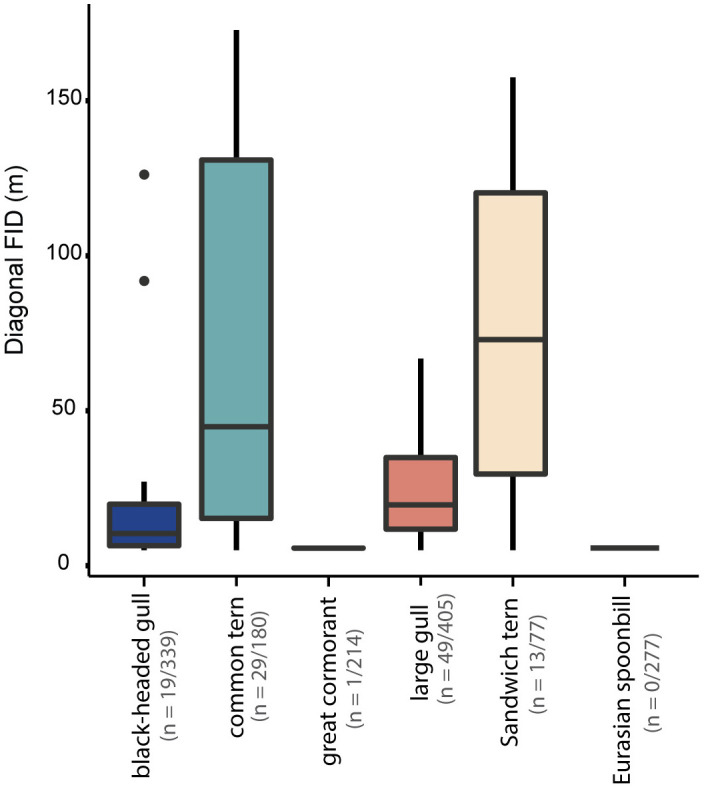
Diagonal FID (m) per species. Diagonal FID (m) is the distance from the location of the drone, accounting for altitude (see methods), to the nearest edge of the colony when a disturbance occurred. Disturbances that occur within the colony are given an FID of 0 **m.** The number of flights (replicates) differs per species, because the number of replicates depended on the number of disturbances that occurred.

Diagonal flight initiation distance (FID), differed significantly between species (Fig 6, Kruskal-Wallis, FID *df* = 3, χ^2^ = 10.41, p = 0.01, see S6 Table for pairwise comparisons). common terns and Sandwich terns had the most sensitive response to drone presence, with a maximum diagonal FID of 173 m and 158 m, respectively. Black-headed gulls and large gulls, on the other hand, had a lower maximum diagonal FID of 126 m and 67 m, respectively (Table 3). We found no significant relationship between diagonal FID and disturbance intensity (S3 Fig.). Moreover, we found no significant relationship between colony composition, i.e., single species vs. mixed species and diagonal FID (Kruskal-Wallis, *df* = 1, χ^2^ = 0.17, p = 0.67; S4 Fig.).

**Table 3:** Minimum, maximum, and mean horizontal and diagonal FID for the different species. Upper 95% confidence intervals were calculated from the mean. The minimum and maximum distances for great cormorants are the same because we only have one disturbance observation for this species. There were no disturbance observations for Eurasian spoonbills. Decimal values were rounded to the nearest whole number.

	Horizontal FID (m)				Diagonal FID (m)			
	Min	Max	Mean	CI (95%)	Min	Max	Mean	CI (95%)
black-headed gull	0	116	17	37	5	126	26	47
large gulls	0	54	13	17	5	67	25	30
Sandwich tern	0	150	68	108	5	158	78	116
common tern	0	165	57	88	5	173	68	97
great cormorant	6	6	–	–	6	6	–	–
Eurasian spoonbills	–	–	–	–	–	–	–	–

## Discussion

Drone-wildlife interactions are increasing globally [34]. Here, we provide species-specific data on safe drone operating distances near coastal breeding bird colonies. We evaluated disturbance in terms of flight initiation distance (FID), number of disturbance events (% of disturbance observations), and intensity (% of birds airborne during events). Flight initiation distance (FID) was species-specific, with Sandwich terns and common terns having the largest FID, suggesting, thus, that these species have the highest sensitivity to drone disturbance, followed by black-headed gulls. Larger gulls were among the least sensitive species to drone disturbance, followed by great cormorants and Eurasian spoonbills, which had the shortest FID and hardly responded to the presence of a drone, even at a < 10m altitude. Additionally, we found no difference in disturbance effects between professional-grade (DJI M210) and consumer-grade (DJI Air) multirotor drones for the seven colony-breeding species that we included in our study. Finally, we found that disturbance generally decreases with increasing altitude. Our results are a step towards best-practice guidelines for research, monitoring, and recreational drone use around colonial breeding birds.

### Species-specific sensitivity and the role of body size

Species varied markedly in their sensitivity to drones, which appears closely linked to body size and perceived predation risk. For instance, Sandwich terns and common terns (small-bodied species) exhibited the largest FID (157–173 m) and were frequently disturbed. In contrast, black-headed gulls were moderately sensitive, and large gulls, great cormorants, and Eurasian spoonbills showed little to no flight response – even at altitudes <10.

These results align with previous studies [16,25,35,36], indicating species-specific differences in disturbance responses. Our results are consistent with previous studies that show that the presence of drones affects species differently. For example, Barr and colleagues, show that drone surveys led to more disturbance in some waterbird species than others [25], with 4 out of the 9 species tested showing significantly higher disturbance scores during drone surveys vs. non-survey periods. In another study, Weston and colleagues, show that bird species varied substantively in FID in response to drone flights (e.g., 8 m in crested pigeon *Ocyphaps lophotes* vs 65 m in little raven *Corvus mellori*) [9]. Similarly, Brisson-Curadeau and colleagues who surveyed four species of Arctic cliff-nesting seabirds and found significantly different behavior for the four species in response to drone flights [35]. Susceptibility may be influenced by body size and predation risk: smaller birds generally have more aerial predators, potentially perceiving drones as threats [14,36]. This is also confirmed by Brisson-Curadeau et al., 2023 [14], who also found that especially smaller species such as shorebirds and terns were most likely to react to drones compared to medium-sized species such as gulls and ducks. Thus, the effect of drones on disturbance is species-specific. Therefore, knowledge of species-specific sensitivity to disturbance is crucial for responsible drone use.

However, not all variation can be explained by size alone. For example, one of the most surprising results in our study was the lack of response of both Eurasian spoonbills and great cormorants. Both species showed minimal flushing in response to drone flights, even at very low (5 and 10 m) altitudes (Fig 5). As these are larger-bodied bird species, their perceived predation risk may be lower compared to smaller species such as terns [14]. However, habituation to drones could also play a role. Several studies found that after an initial drone flight, birds displayed fewer adverse behaviors during subsequent passes, or birds returning to their starting positions after being flushing while the cause of disturbance – a drone – was still airborne [35–38]. However, except for habituation within our study, we have no reason to expect bird habituation to drones within the study areas in general. Some colonies have been exposed to drones in previous years (S2 Table, the baseline study) to collect baseline data, but the wider Wadden Sea is a World Heritage Site and a Natura 2000 area where drone flights are generally restricted. Some colonies may be more habituated to human disturbance in general due to their proximity to roads and inhabited areas (Texel, Buitenheim-Zuid). In contrast, others were more remote (e.g., Griend). Thus, body size offers an explanatory framework but should not be used as a sole predictor of species-sensitivity to drones.

### Colony composition and size

Beyond species traits, the social context, such as colony composition and size, also affects a species’ response to drones. Colonies with one sensitive species may cascade into collective disturbance [39,40]. Although we could only test colony composition for large gulls (found in both single and mixed colonies), no difference in FID was detected. This might be due to co-occurring Eurasian spoonbills, a species with low sensitivity. A comparison containing one sensitive species (e.g., common terns or Sandwich terns) and a less sensitive species would be ideal to test this. Furthermore, the presence of non-breeding species such as, oystercatchers, which are known to be highly sensitive to disturbance [41] could also have cascading effects on breeding birds. In addition to the species composition of the colony, colony size can also have an impact on disturbance, with larger colonies being more likely to respond to an approaching drone at larger distances [1,26]

### Undetected or subtle disturbance

It is important to note that the absence of visible disturbance does not imply the absence of impact. Some species (great cormorants, Eurasian spoonbills) did not show overt behavioral (flushing) responses. However, Several studies suggest that disturbance can manifest physiologically, e.g., elevated heart rates and/or corticosterone levels without obvious behavioral response [2,42–44]. In our study, we defined disturbance as a deviation from ‘baseline’ behavior, and we determined that disturbance took place when > 10% of the colony birds were airborne. However, there is no standard definition of disturbance, and it is defined differently across studies. This is probably partly due to differences in behavioral displays. Therefore, our findings may underestimate disturbance, and future research should integrate physiological measurements.

### Flight altitude

Another key factor influencing disturbance is flight altitude. We found a negative relationship between altitude and percentage of disturbance observations for black-headed gulls, common terns, and larger gulls, with a higher percentage of disturbance observations at lower altitudes. The majority of disturbance in these cases led to 10–50% of the colony being airborne. The relationship between drone flight altitude and a behavioral disturbance response has been reported in other studies [15] such as Mesquita and colleagues, who show that with decreasing diagonal distance (equivalent to lower altitude), the percentage of disturbance in two species of swifts increases [32]. Moreover, Weimerskirch and colleagues tested the effect of drones on different sub-Antarctic seabirds species at comparable altitudes to the ones in our study [45], and found similar results as ours, with an increase in behavioral responses when the altitude of the drone decreased. Interestingly, Weimerskirch and colleagues included the imperial cormorants (*Phalacrocorax atriceps*) in their study, and found them to be among the most sensitive species to drone disturbance [45], contrary to what we conclude. This difference could be attributed to species-specific responses, as we tested great cormorants and Weimerkirch and colleagues [45] tested imperial cormorants. Alternatively, our conclusions might differ because Weimerkirch and colleagues recorded a range of behavioral responses along a spectrum of disturbance, including ‘vigilance’ and ‘looking at the drone’. In contrast, we determine disturbance as a flight-response only. We acknowledge that disturbance can be displayed in different ways at different levels, which means that the conclusions we make based on flight responses alone might underestimate disturbance effects.

Furthermore, we did not find a relationship between altitude and disturbance behavior for Sandwich terns, probably because of methodological constrains. Since we were unable to fly over Sandwich tern colonies at altitudes ≤ 20 m because flights at 20 m always caused a disturbance, our sample size for Sandwich terns is likely too small to show an effect of altitude. While we cannot conclude with certainty that Sandwich terns follow the same trends as black-headed gulls, common terns and larger gulls, our data suggests that Sandwich terns are highly sensitive to drone disturbance. It is possible that if we had followed a different protocol where flights were carried out at lower altitudes despite causing disturbance at altitudes above the disturbance point, we would have found a significant effect of altitude on the percentage of disturbance observations.

### Drone type and flight characteristics

Apart from altitude, the characteristics of the drone and flight style may also influence disturbance, with drones that look more like aerial predators producing more disturbance [7,16,46–50]. We did not find a difference in disturbance between a small consumer drone (0.6 kg) and a larger professional multirotor (4.3 kg), likely because they had similar shapes and noise profiles and resembled aerial predators minimally. However, fixed-wing drones might provoke stronger responses [47], and should be included in future tests.

Other characteristics, such as size, sound intensity and angle of approach, may influence detection and perception of threat [51]. Larger drones possibly elicit reactions at greater distances because the size of the ‘threat’ could increase the perceived risk [52], and the probability of detecting it [1]. Moreover, drone noise and the increase in sound pressure level with changes in speed or altitude are factors that could affect disturbance behavior [7,48–50]. However, the effect of noise is difficult to quantify since it varies with many other factors such as, the importance of acoustic cues for the studied species (e.g., whether predators are detected acoustically), the altitude of the drone, the sound propagation characteristics of the environment and the background noise levels [53]. Moreover, the angle of approach can also have a marked impact on species, with vertical approaches being more disturbing due to their similarity to a predator’s attack [50], but see [35]. While we followed a target-oriented approach (representative of professional drone use [1]), the potential impact of the choices listed above should be considered when interpreting our findings.

### Limitations

While our study provides valuable insights, there are also limitations to consider. This study addresses the behavioral disturbance effects of drones on colony-breeding shorebirds. While different species have been included in the study – terns, gulls, cormorants, and spoonbills – these results are specific to colonial breeding birds and cannot be generalized to solitary breeding birds, passerines or other wildlife. Additionally, these results are specific to a certain life stage of these birds: the breeding season. We did not test the effects of drones on other types of behavior of these bird species, such as foraging and roosting, and more research is needed to address this. Furthermore, our experimental design required us to stop the disturbance trials for a given colony after several disturbance events had occurred. Additionally, we did not have access for the same number of colonies for all species. As a result, we obtained a smaller and uneven sample size for some species (e.g., terns, cormorants, spoonbills), which reduced the statistical power of our models. Therefore, the results for these species should be interpreted with caution.

### Recommendations for management and best practices

Based on our findings, we propose the following guidelines to minimize drone-related disturbance. The use of drones in nature is growing rapidly for both professional and recreational use, and policy and guidelines are lagging behind despite the mounting evidence of the disturbing effects of drones on wildlife [1,2,16,25,46,47,50,54]. However, there is an enormous potential to use drones for monitoring, research and wildlife conservation [55–58]. Hence, there is an urgent need to develop best-practice guidelines for nature managers, policymakers, and researchers. In this study, we show that drone disturbance by multi-rotor drones was generally low for the 7 studied colonial bird species: great cormorant, Eurasian spoonbill, herring gull, lesser black backed gull, Sandwich tern, and common tern. However, the smaller species (terns) were generally more sensitive to disturbance than the other species. Based on our results regarding flight initiation distance (FID), we recommend the following safe operating (diagonal) distances of drones near colonial shorebird species (Table 4): > 170 and 160 m for common and Sandwich tern, respectively, > 110 m for black-headed gulls and >50 m for large gulls, Eurasian spoonbills, and great cormorants. We recommend starting an approach to a colony at a minimum flight altitude of 50 m, and staying at or above this altitude when flying for the first time over a colony. Additionally, sensitivity to drone disturbance by the most sensitive species should be considered for mixed colonies, e.g., when flying in colonies with Sandwich terns or common terns. The distances we identified in this study can be used to develop policy and management guidelines for the use of drones in breeding areas.

**Table 4:** Recommendations of no-fly zones for the colonies of common terns, Sandwich terns, black headed gulls, herring gulls and lesser black-backed gulls, great cormorants and Eurasian spoonbills. The horizontal distance refers to the horizontal flight distance limit to the colony. The altitude indicates a general minimum safe altitude we recommend for all species, but see recommendations above for case-specific situations and special considerations.

Species	Horizontal distance (m)	Altitude (m)
common tern	170	50
Sandwich tern	160	50
black-headed gull	110	50
large gulls	50	50
great cormorant	50	50
Eurasian spoonbill	50	50

Since drones are also a growing tool for wildlife monitoring and conservation, our results may also be used to determine optimal flight altitudes for research and monitoring. For most species, an altitude of >50 m would be an optimal choice for mapping and counting purposes, especially because optics have improved considerably, reducing the altitude needed for monitoring purposes. However, if necessary, flight altitude can be as low as 10 m for great cormorants and Eurasian spoonbills. Other recommendations for responsible drone flights across seabird colonies (not derived from this study) include 1) flying in a lawn-mower pattern [1], as it can be perceived less as an attack, 2) a gradual, horizontal approach for target-oriented flights [50], 3) a minimum take-off distance that is > 200 m depending on local logistics and drone flight rules for line of sight, 4) check for the presence of vigilant and potentially aggressive or sensitive species such as oystercatchers, avocet and curlews and consider postponing the flight to avoid potentially cascading effects due to the behavioral response of these species and finally, 5) each flight should be evaluated, and when a disturbance is caused, with a > 10% flight behavior, we suggest to terminate the flight, to allow sufficient recovery time before the next attempt.

In addition to seeking species-specific guidance for drone use around wildlife, we suggest establishing or following a protocol for drone-based data collection and analysis to ensure a streamlined and rigorous development of scientific products. For example, [59] make an overview of activities that should be involved in drone-collected data, including a flight mission, followed by a clear study design and pre-flight fieldwork for a reconnaissance of the area that will be surveyed.

## Supporting information

S1 Text Determining baseline flight behavior in shorebird colonies. (PDF)

S1 Table Colony information. Names, species found on the colonies and the coordinates.
(PDF)

S2 Table Baseline levels of % birds airborne in coastal breeding bird colonies as indicated by mean % of birds airborne, the range determined across colonies and the number of colonies measured.
(PDF)

S3 Table Example of a flight series. Air is short for DJI Air 2S and M210 is short for DJI Matrice 210. The first row under the flight altitude column (1–7) does not represent a progressive order of altitude per flight. It serves to identify the flight number within a flight series. Flight altitude per series was randomized, with the exception of 5 m, which was never used as an initial altitude. Drone type was randomized between flight series.
(PDF)

S4 Table Pairwise comparison of disturbance between species. Differences were calculated with a Wilcoxon test. Bold font indicates a statistically significant difference (P value).
(PDF)

S5 Table Confidence Intervals (CI) calculated on multinomial model. The model tested the interaction between species and altitude on disturbance at different intensities. The lower limit is 2.5% and the upper limit is 97.5%.
(PDF)

S6 Table Pairwise comparisons between species for diagonal FID. Differences were calculated by a pairwise Wilcox test.
(PDF)

S1 Fig Example of drone disturbance experiment flight. Flights were conducted in a “target-oriented” manner, approaching the colony horizontally at different altitudes. Map data from OpenStreetMap.
(TIF)

S2 Fig Number of flights per drone type, per species, per altitude. (TIF)

S3 Fig Horizontal and diagonal FID per disturbance intensity category, per species. There was no significant relationship between disturbance intensity category and horizontal or diagonal FID (m).
(TIF)

S4 Fig Differences in diagonal and horizontal FID for large gulls in mixed- and single- species colonies. There was no significant relationship between colony type (mixed vs. single) and horizontal or diagonal FID (m).
(TIF)
