# Supplementary material for: Best practice guidance for recreational and professional drones near colonial breeding birds
Source: PLoS One. 2025 Nov 5;20(11):e0332619. doi: 10.1371/journal.pone.0332619 (PMC12588502; doi:10.1371/journal.pone.0332619)
Supplement: S1 Text — (PDF) [file pone.0332619.s001.pdf]

S1 Text. Determining baseline flight behavior in shorebird colonies

We recorded the behavior of colonial coastal breeding birds in different colonies to study the baseline levels of airborne bird levels as a reference for behavioral flight response due to drone disturbance. For this, we visited 11 locations with 14 colonies of 6 different bird species; spoonbill (n=3), black-headed gull (n=4), herring gull and lesser black-backed gulls (grouped as large gulls) (n=2), sandwich tern (n=2), common tern (n=3) in the breeding season of 2020. All colonies were in the Dutch Wadden Sea area. These colonies were observed for about 6 hours around low tide (-/+ 3h around low tide) to prevent potential additional disturbance of roosting shorebirds during high tide. We were also restricted to low-tide flights due to permit requirements. Two independent trained observers observed bird behavior at a 5-minute interval and estimated the number of birds airborne above the colony at that particular time. These numbers were averaged, and % birds airborne was calculated based on counts of the actual total bird numbers in the breeding colony, counted on drone images. Drone flights or other disturbance events were excluded from this dataset. Thus, depending on species and colony, we could determine the 'baseline' flight activity within colonies, which ranged between 0-12%. (Table S2). Based on these data, we only considered behavioral flight response due to drone disturbance >10% as a deviation from the baseline which we consider 'normal behavior' and thus 'disturbance'.
