# Supplementary material for: Best practice guidance for recreational and professional drones near colonial breeding birds
Source: PLoS One. 2025 Nov 5;20(11):e0332619. doi: 10.1371/journal.pone.0332619 (PMC12588502; doi:10.1371/journal.pone.0332619)
Supplement: S1 Table — Names, species found on the colonies and the coordinates. (PDF) [file pone.0332619.s002.pdf]

Table S1. Colony information. Names, species found on the colonies and the coordinates

Colony	Colony ID	Mixed colony	Species	Zoned	N (monitored)	N (total)	Monitored area size (m^2)	Total area size (m^2)	Coordinates	Take-off distance
Texel – Buitenheim-Zuid island 1	1	No	Black headed gulls	Yes	314	582	1116.2	2220.5	53°03'20.6"N 4°51'58.7"E	120 m
Texel – Buitenheim-Zuid island 2	2	Yes	Black headed gulls	No	–	41	–	979.4	53°03'17.0"N 4°52'08.8"E	350 m
			Common tern		–	51				
Texel – De Muy	3	Yes	Spoonbills	Yes	11	11	34263.5	6310.2	53°07'16.4"N 4°47'00.2"E	215 m
			Cormorants		129	141				
Texel – De Geul	4	No	Large gulls	Yes	58	74	3144.8	4511.6	53°01'01.9"N 4°42'48.9"E	85 m
Vlieland – Kroon’s polder	5	Yes	Large gulls	No	–	42	–	1776.9	53°15'23.7"N 4°58'13.3"E	200 m
			Spoonbills		–	17				200 m

Vlieland – Kroon’s polder island	6	No	Cormorants	No	–	210	–	689.3	53°15'23.7"N 4°57'42.7"E	420 m
Terschelling – De Groede	7	No	Large gulls	Yes	54	163	5429.2	10882.7	53°24'43.7"N 5°25'56.4"E	240 m
Griend – Inside Dike	8	Yes	Common terns	No	–	100		256.7	53°15'05.3"N 5°15'00.1"E	300 m
			Sandwich terns		–	329		105.48	53°15'04.8"N 5°15'00.6"E	300 m
Griend - Dike	9	No	Black headed gulls	Yes	250	400		1958.2	53°15'02.8"N 5°14'58.3"E	230 m
Mainland – Punt van Reide	10	No	Black headed gulls	No	–	385		1074.1	53°18'25.3"N 7°05'48.7"E	170 m
