# Supplementary material for: Best practice guidance for recreational and professional drones near colonial breeding birds
Source: PLoS One. 2025 Nov 5;20(11):e0332619. doi: 10.1371/journal.pone.0332619 (PMC12588502; doi:10.1371/journal.pone.0332619)
Supplement: S2 Table — measured. (PDF) [file pone.0332619.s003.pdf]

**Table S2** Baseline levels of % birds airborne in coastal breeding bird colonies as indicated by mean % of birds airborne, the range determined across colonies and the number of colonies measured.

| | Mean (%) | Range (%) | nº Colonies |
|---|---|---|---|
| Eurasian Spoonbill | 0 | 0-1 | 3 |
| Black headed gull | 3 | 1-5 | 4 |
| Large gulls | 4 | 4-5 | 2 |
| Common tern | 10 | 8-12 | 2 |
| Sandwich Tern | 4 | 2-6 | 3 |
