# Supplementary material for: Best practice guidance for recreational and professional drones near colonial breeding birds
Source: PLoS One. 2025 Nov 5;20(11):e0332619. doi: 10.1371/journal.pone.0332619 (PMC12588502; doi:10.1371/journal.pone.0332619)
Supplement: S3 Table — Air is short for DJI Air 2S and M210 is short for DJI Matrice 210. The first row under the flight altitude column (1–7) does not represent a progressive order of altitude per flight. It serves to identify the flight number within a flight series. Flight altitude per series was randomized, with the exception of 5 m, which was never used as an initial altitude. Drone type was randomized between flight series. (PDF) [file pone.0332619.s004.pdf]

Table S3 Example of a flight series. Air is short for DJI Air 2S and M210 is short for DJI Matrice 210. The first row under the flight altitude column (1-7) does not represent a progressive order of altitude per flight. It serves to identify the flight number within a flight series. Flight altitude per series was randomized, with the exception of 5 m, which was never used as an initial altitude. Drone type was randomized between flight series.

Location	Day	Drone type	Flight number	Flight altitude						
				1	2	3	4	5	6	7
Texel	1	Air	1	20	10	40	30	50	5	15
Texel	1	M210	2	30	15	40	20	50	5	10
Texel	1	Air	3	10	30	40	5	20	50	15
Texel	1	Air	4	30	40	15	50	5	10	20
Texel	1	Air	5	30	15	20	40	5	10	50
Texel	1	M210	6	20	20	50	5	10	30	15
Texel	1	M210	7	20	40	5	30	10	15	50
Texel	1	M210	8	20	50	10	15	30	20	5
