# Supplementary material for: Best practice guidance for recreational and professional drones near colonial breeding birds
Source: PLoS One. 2025 Nov 5;20(11):e0332619. doi: 10.1371/journal.pone.0332619 (PMC12588502; doi:10.1371/journal.pone.0332619)
Supplement: S4 Table — Differences were calculated with a Wilcoxon test. Bold font indicates a statistically significant difference (P value). (PDF) [file pone.0332619.s005.pdf]

Table S4. **Pairwise comparison of disturbance between species.** Differences were calculated with a Wilcoxon test. Bold font indicates a statistically significant difference (P value)

	Eurasian spoonbill	large gulls	great cormorant	black-headed gull	common tern
large gulls	**5.1e-08**	-	-	-	-
great cormorant	0.21067	**6.1e-07**	-	-	-
black-headed gull	**0.00146**	**0.00019**	**0.01311**	-	-
common tern	**2.2e-06**	0.48843	**2.7e-05**	**0.01266**	-
Sandwich tern	**9.5e-07**	0.86855	**1.6e-05**	**0.00746**	0.52234
