# Supplementary material for: Best practice guidance for recreational and professional drones near colonial breeding birds
Source: PLoS One. 2025 Nov 5;20(11):e0332619. doi: 10.1371/journal.pone.0332619 (PMC12588502; doi:10.1371/journal.pone.0332619)
Supplement: S5 Table — The model tested the interaction between species and altitude on disturbance at different intensities. The lower limit is 2.5% and the upper limit is 97.5%. (PDF) [file pone.0332619.s006.pdf]

Table S5. Confidence Intervals (CI) calculated on multinomial model. The model tested the interaction between species and altitude on disturbance at different intensities. The lower limit is 2.5% and the upper limit is 97.5%

	**10-50%**		**50-75%**		**75-100%**	
	**lwr**	**upr**	**lwr**	**upr**	**lwr**	**upr**
**Intercept**	-2.167	-1.288	-3.346	-1.968	-5.652	-2.748
**black-headed gull**	-0.108	-0.034	-0.092	-0.005	-0.135	0.042
**large gulls**	-0.048	-0.008	-0.91	-0.008	-0.111	0.038
**Sandwich tern**	-0.025	0.012	-0.054	0.019	-0.065	0.053
**common tern**	-0.023	0.009	-0.026	0.021	-0.093	0.050
**cormorant**	-0.743	-0.045	-18.801	15.477	-23.652	20.739
**spoonbill**	-18.477	14.696	-18.138	14.736	-26.792	23.664
