# Supplementary material for: Best practice guidance for recreational and professional drones near colonial breeding birds
Source: PLoS One. 2025 Nov 5;20(11):e0332619. doi: 10.1371/journal.pone.0332619 (PMC12588502; doi:10.1371/journal.pone.0332619)
Supplement: S6 Table — Differences were calculated by a pairwise Wilcox test. (PDF) [file pone.0332619.s007.pdf]

**Table S6. Pairwise comparisons between species for diagonal FID.** Differences were calculated by a pairwise Wilcox test.

| | Diagonal disturbance distance (P value) | | |
|---|---|---|---|
| | black-headed gull | common tern | large gulls |
| common tern | 0.085 | - | - |
| large gulls | 0.142 | 0.092 | - |
| Sandwich tern | 0.085 | 0.788 | 0.085 |
