# Supplementary figures and images for: Best practice guidance for recreational and professional drones near colonial breeding birds
Source: PLoS One. 2025 Nov 5;20(11):e0332619. doi: 10.1371/journal.pone.0332619 (PMC12588502; doi:10.1371/journal.pone.0332619)

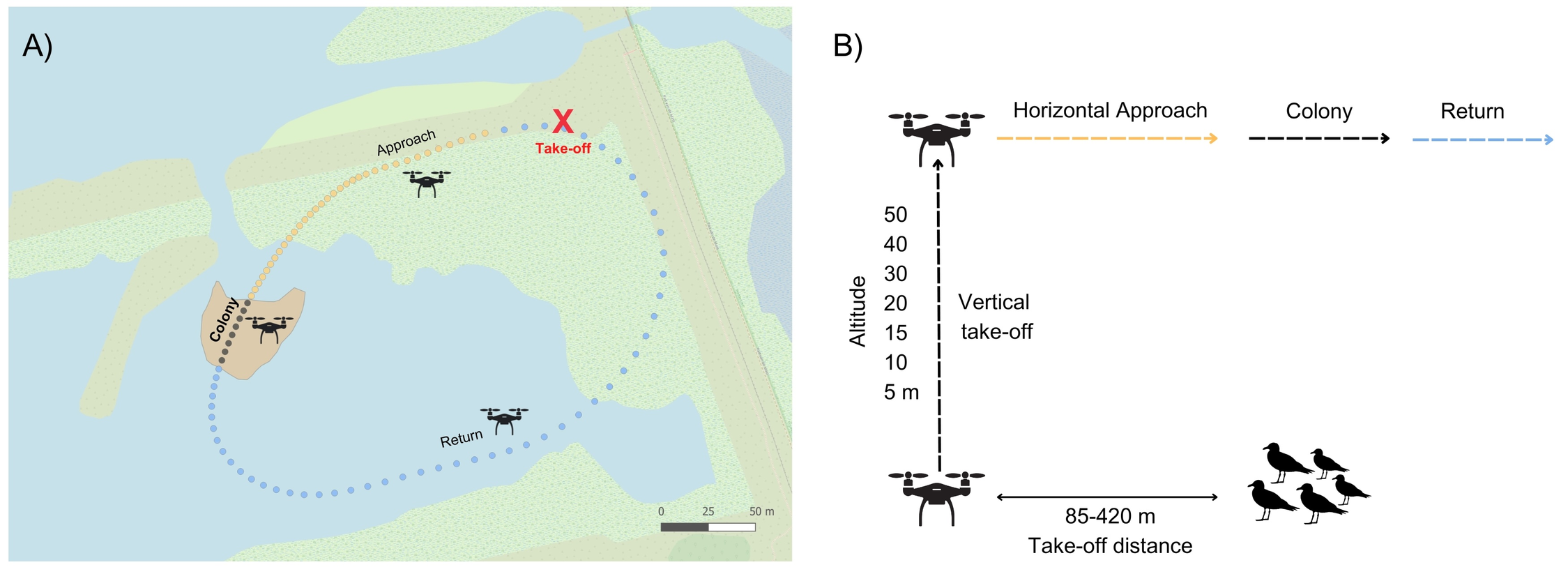

Supplement: S1 Fig — Flights were conducted in a “target-oriented” manner, approaching the colony horizontally at different altitudes. Map data from OpenStreetMap. (TIF) [file pone.0332619.s008.tif]

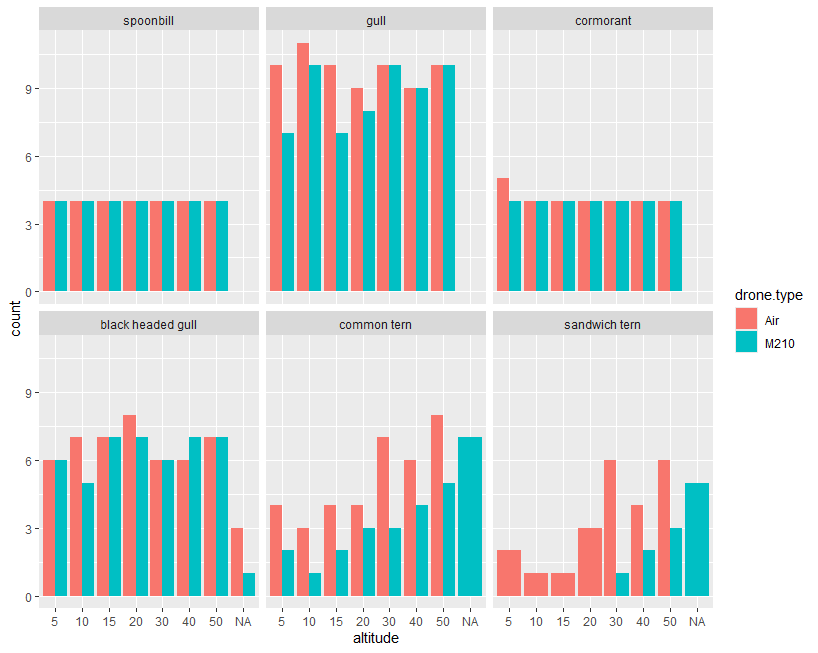

Supplement: S2 Fig — (TIF) [file pone.0332619.s009.tif]

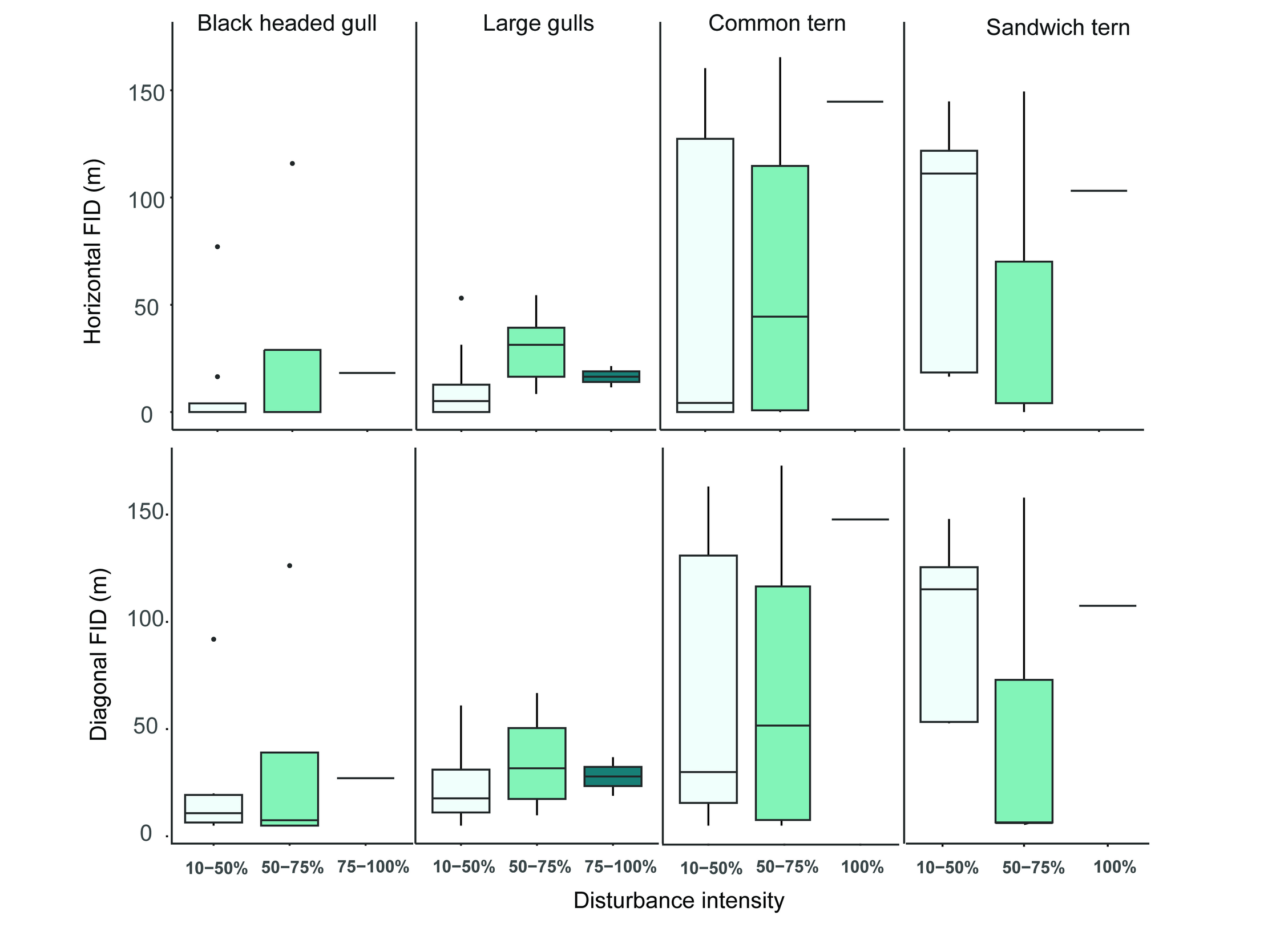

Supplement: S3 Fig — There was no significant relationship between disturbance intensity category and horizontal or diagonal FID (m). (TIF) [file pone.0332619.s010.tif]

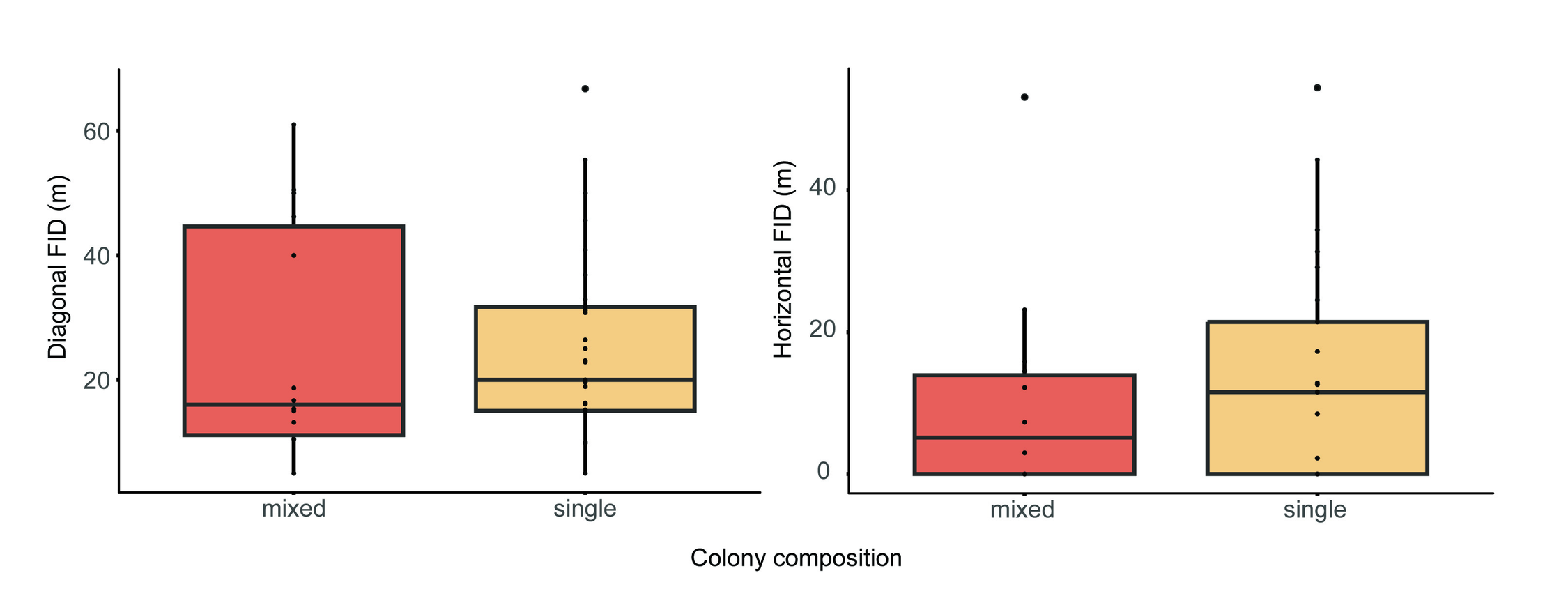

Supplement: S4 Fig — There was no significant relationship between colony type (mixed vs. single) and horizontal or diagonal FID (m). (TIF) [file pone.0332619.s011.tif]
